# Detection of heartworm antigen without cross-reactivity to helminths and protozoa following heat treatment of canine serum

**DOI:** 10.1186/s13071-020-04573-6

**Published:** 2021-01-22

**Authors:** Jeff M. Gruntmeir, Nina M. Thompson, Maureen T. Long, Byron L. Blagburn, Heather D. S. Walden

**Affiliations:** 1 Department of Comparative, Diagnostic and Population Medicine, University of Florida College of Veterinary Medicine, 1945 SW 16th Avenue, Gainesville, FL 32610 USA; 2 Department of Pathobiology, Auburn University College of Veterinary Medicine, 1130 Wire Road, Auburn, AL 36849 USA

**Keywords:** *Dirofilaria immitis*, *Acanthocheilonema reconditum*, Heat treatment, Intestinal parasites, Microfilariae, Cross reactivity, Antigen

## Abstract

**Background:**

Detection of *Dirofilaria immitis*, or heartworm, through antigen in sera is the primary means of diagnosing infections in dogs. In recent years, the practice of heat-treating serum prior to antigen testing has demonstrated improved detection of heartworm infection. While the practice of heat-treating serum has resulted in earlier detection and improved sensitivity for heartworm infections, it has been suggested that heat treatment may cause cross reactivity with *A. reconditum* and intestinal helminth infections of dogs. No studies have assessed the potential cross-reactivity of these parasites with heartworm tests before and after heat treatment using blood products and an appropriate gold standard reference.

**Methods:**

Canine sera (*n*=163) was used to evaluate a heartworm antigen-ELISA (DiroCHEK®) and potential cross-reactivity with common parasitic infections. The heartworm status and additional parasite infections were confirmed by necropsy and adult helminth species verified morphologically or by PCR, and feces evaluated by centrifugal fecal flotation.

**Results:**

Intestinal parasites were confirmed in 140 of the dogs by necropsy, and 130 by fecal flotation. *Acanthocheilonema reconditum* microfilariae were confirmed in 22 dogs. Prevalence of heartworm infection confirmed by necropsy was 35.6% (58/163). In the 105 dogs without heartworms, specificity remained unchanged at 100% both before and after heat treatment despite confirmed infections with *A. reconditum*, *Ancylostoma caninum*, *Ancylostoma brasiliense*, *Trichuris vulpis*, *Toxocara canis*, *Dipylidium caninum*, *Spirometra mansonoides*, *Macracanthorynchus ingens*, *Cystoisospora* sp., *Giardia* sp., and *Sarcocystis* sp.

**Conclusions:**

These findings suggest that the use of heat treatment improves sensitivity of heartworm tests and is unlikely to cause false positive antigen results due to *Acanthocheilonema reconditum*, intestinal helminths, and protozoal parasites in dogs.

**Graphic abstract:**

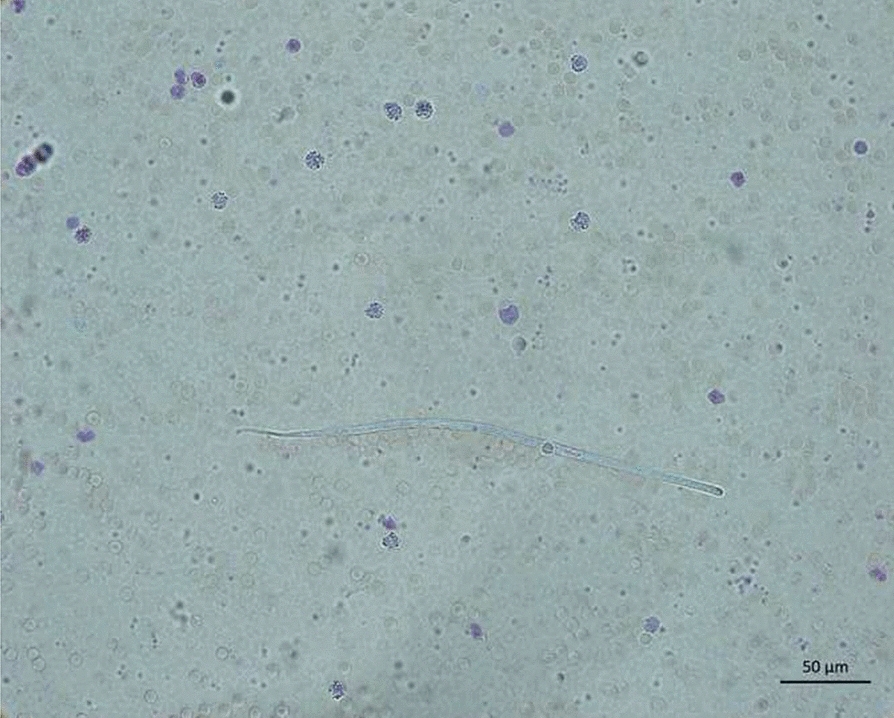

## Background

The use of antigen testing to aid in the diagnosis of canine heartworm infections has been a vital tool for veterinarians since 1985. Commercialized antigen tests, primarily based on monoclonal antibodies generated from adult heartworm antigen preparations following acid and heat purification [1], or other identified non-cross-reactive antigens [2], demonstrated high specificity [3–5]. The relative high specificity of commercial *Dirofilaria immitis* antigen tests is in contrast to the cross-reactive nature of antibody tests historically used for *D. immitis* [6, 7] and more recently described as a diagnostic approach examining human exposure to *Dirofilaria* sp. [8, 9] and potential application for *D. repens* infected dogs [10].

Prior to 1995, manufacturer protocols for many commercial heartworm antigen tests used chemical and/or low heat (60–70Cº) immune complex dissociation (ICD) steps. However recently heat ICD treatment of serum at an elevated temperature of 104 Cº has been shown to allow earlier detection and improved sensitivity for *D. immitis* antigen [3]. No decrease in specificity has been observed in pathogen free dogs when using this elevated heating step or an acid ICD prior to antigen testing [3, 11]. Heat ICD is also unlikely to cause false positive results on heartworm tests in dogs infected by *O. lupi* [13].

Studies evaluating commercial antigen test specificity have used blood products from dogs experimentally or naturally infected with *Acanthocheilonema reconditum, Dirofilaria repens, Onchocerca lupi, Toxocara canis, Ancylostoma caninum, Ancylostoma brasiliense, Uncinaria stenocephala, Trichuris vulpis, Dipylidium caninum, Strongyloides stercoralis, Spirocerca lupi* and *Angiostrongylus vasorum* [1, 4, 6, 12–19, 21]. In most instances, no cross reactivity with antigen tests was observed using serum from dogs both with or without the historical ICD steps [1, 4, 6, 12–16, 19], however two commercially available tests were shown to cross-react with *A. vasorum* [17], and three demonstrated cross-reactivity with *S. lupi* [18]. In a rare case, an exceptionally high infection intensity by *Acanthocheilonema dracunculoides*, induced a false antigen positive result using normal manufacturer protocols [20]. *Dirofilaria repens* has recently been confirmed to cross-react with 3 heartworm tests following heat ICD in a small number of experimentally infected n=3 dogs [21]. Although some but not all cases of naturally infected, patent *D. repens* infections, have positive antigen results post-heat ICD [22].

Several recent publications have added confusion to the literature by stating *Acanthocheilonema reconditum* causes false positives with heartworm tests [23–27] misciting a study using a non-commercialized ELISA based antibody test [7], or based on interpretation of study results using an inappropriate gold standard reference (PCR results) [26], which can neither verify nor rule out an occult (amicrofilaremic) *D. immitis* infection or *D. repens* infection. Additionally, conclusions of studies using non-biologically relevant samples, such as saline soakings of intestinal helminths, to assess cross-reactivity with heartworm tests, are potentially misleading [23]. Currently no published studies suggesting cross reactivity of *A. reconditum* or intestinal helminths with heartworm antigen tests, with or without heat ICD of naturally sourced canine serum, have ruled out occult *D. immitis* infection by necropsy.

In this study, using sera from 163 dogs, 100 of which were previously characterized based on heartworm composition (adults and microfilariae) and antigen results with and without heat ICD [28], we assessed heartworm antigen detection in relation to potential cross-reactivity with *A. reconditum*, intestinal helminths and protozoan infections using a commercially available heartworm antigen test.

## Methods

From 2017 to 2020, 163 previously euthanized dogs were collected and necropsied at the University of Florida College of Veterinary Medicine. All dogs collected during this time period were included in this study. The abdominal and thoracic cavities were thoroughly inspected for parasites prior to and following removal of the heart, lungs, caudal esophagus, thoracic aorta, and gastrointestinal (GI) tract. The entire GI tract was opened and mucosa scraped and rinsed into a 355 µm sieve for parasite recovery. Helminths were rinsed in phosphate buffered saline (PBS) and stored in 70% ethanol. Feces removed from the colon was examined by centrifugal flotation using Sheather’s sugar (SG 1.26), and when necessary, by acid fast staining (i.e. *Cryptosporidium* verification) [29]. Antigen testing was completed using the DiroCHEK® assay (Zoetis LLC, Parsippany, NJ) both before and after heat ICD of serum [3]. Heat ICD was performed with a reduced starting serum volume of 400 µl [28].

Whole blood from all dogs was examined for microfilariae by direct smear and Modified Knott’s technique (MKT). Microfilariae recovered from whole blood by MKT were identified morphometrically [3, 29, 30]. Microfilariae from whole blood samples were processed for DNA extraction (DNeasy Blood and Tissue kit, Qiagen) [31], modified by using elution buffer heated to 70 °C. Microfilariae species were confirmed by conventional PCR, specific for *D. immitis* (12S rDNA) and *D. repens* (12S rDNA), using single-plex reactions to increase sensitivity [32]. Specific PCR for *A. reconditum* (cox1) was performed as described [31] modified by using 54 °C as the optimal annealing temperature. A Filariid-generic PCR assay targeting cox1 and 12S rDNA genes were also used for mono-infections [31].

The prevalence (PR), sensitivity (SE), specificity (SP), negative (NPV) and positive predictive values (PPV), and 95% confidence intervals (“exact” Clopper-Pearson) were calculated for antigen results using a diagnostic test 2x2 contingency table using a commercial statistical software (MedCalc Statistical Software version 19.1.2). The McNemar paired *χ*^2^ test was used to compare SE and SP before and after heat treatment of serum and for the calculation of two sided *p*-values, with *P*<0.05 considered statistically significant [33]. The PPV, the probability that the disease is present when the test is positive, was calculated as SE × PR / [SE × PR + (1−SP) × (1−PR)]. The NPV, the probability that the disease is absent when the test is negative, was calculated as SP × (1−PR) / [(1−SE) × PR + SP × (1−PR)] [34].

## Results

The presence of *D. immitis* adult or immature adult worms was verified by necropsy. Viable heartworms were found in 58/163 (35.6%) dogs (Table 1). Of those, 51/58 had mature heartworms present (36 mixed sex, 8 female only, 7 male only). Ectopic infections were present in 4 of the 58 *D. immitis* necropsy positive dogs, each with a single live mature heartworm in the thoracic cavity. Only 3 of these 4 dogs with ectopic *D. immitis* also had heartworms recovered from the cardiopulmonary system, 1 of the 4 had only the single *D. immitis* in the thoracic cavity. Additionally, 7/58 had only immature heartworms (3 female and 4 male only). A total of 105 dogs, without viable heartworms present at necropsy, were classified as non-infected. Microfilariae were detected in 38/163 blood samples by the MKT (Table 1); 30/58 heartworm infected dogs and 8/105 heartworm non-infected dogs. A total of 48.3% of heartworm infected dogs had occult infections. Of the 30 MKT positive heartworm infected dogs, 15 blood samples had microfilariae morphologically identified as *D. immitis* only, 1 sample with *D. immitis* and two microfilariae of an unknown species (measuring 427 × 7.6 µm and 408.6 × 7.3 µm), 2 samples had microfilariae of *A. reconditum* only, and 12 with microfilariae of both *D. immitis* and *A. reconditum*. Microfilariae of all 8 MKT positive samples in heartworm non-infected dogs were identified as *A. reconditum* (Table 1). Molecular confirmation of microfilariae (Table 1) included *D. immitis* specific PCR targeting 12S rDNA and amplified all 28 MKT positive samples for *D. immitis* microfilariae. *A reconditum* specific PCR targeting cox1, amplified all 10 MKT positive samples where *A. reconditum* was the only microfilariae detected. The *A. reconditum* cox1 PCR did not amplify any of the 12 samples positive for *A. reconditum* when *D. immitis* was also present. *Dirofilaria repens* was not detected in any samples by MKT or specific 12S rDNA PCR.

**Table 1:** Necropsy, Modified Knott’s technique, DiroCHEK® antigen results with and without heat ICD, sensitivity or specificity, and PCR results for 58 *Dirofilaria immitis* necropsy positive and 105 *D. immitis* necropsy negative dogs

*D. immitis* Necropsy	Modified Knott’s technique (# pos)	DiroCHEK^®^: antigen without heat ICD (#pos/total)	DiroCHEK®: sensitivity or specificity without heat ICD	DiroCHEK^®^: antigen with heat-ICD (#pos/total)	DiroCHEK®: sensitivity or specificity with heat ICD	PCR
*D. immitis* Positive^a,b^ *n*=58	*D. immitis* (15)	15/15	Sensitivity # 69.0% (40/58)	15/15	Sensitivity # 87.9% (51/58)	*D. immitis*
	*D. immitis* + *A. reconditum (12)*	11/12		12/12		*D. immitis + ND*
	*D. immitis* + unk. species^d^ (1)	1/1		1/1		*D. immitis*
	*A. reconditum (2)*	0/2		2/2		*A. reconditum*
	Not detected (27)	13/28		21/28		–
*D. immitis* Negative^c^ *n*=105	*A. reconditum (8)*	0/8	Specificity 100%	0/8	Specificity 100%	*A. reconditum*
	Not detected (97)	0/97		0/97		–

^a^Includes 1 dog with 1 adult female heartworm present in thoracic cavity but none in pulmonary arteries

^b^Includes 7 dogs with only immature heartworms

^c^Includes 1 dog with only embolized heartworm fragments

^d^2 unknown microfilariae species measuring 427.6 × 7.6 µm and 408.6 × 7.3 µm

The sensitivity of the DiroCHEK® before and after heat treatment of sera for *D. immitis* antigen among all heartworm infections (Table 1) was 69.0% (40/58) (95% CI 55.5–80.5%) and 87.9% (51/58) (95% CI 76.7–95.0%) respectively *p*=0.001, and mature heartworms was 78.4% (40/51) (95% CI 64.7–88.7%) and 98.0% (50/51) (95% CI 89.6–99.9%) p=0.002. The observed increases in sensitivity were statistically significant. Altogether, 90.9% of heartworm infected dogs initially testing false antigen negative but post-heat ICD antigen positive were verified as occult, male or female only heartworm infections. All 105 dogs verified as heartworm negative at necropsy tested antigen negative, a specificity of 100%, (95% CI 96.6–100.0%) both before and after heat treatment (Table 1). Considering only mature heartworm infections, the positive predictive value of the DiroCHEK® remained unchanged at 100%, and the negative predictive value improved from 85.4% (95% CI 79.9–89.5%) to 98.9% (95% CI 92.9– 99.9%) following heat treatment.

Of the 163 dogs examined in the study, intestinal helminths were recovered by necropsy in 140 (85.9%) and included species of nematodes (*n*=128), cestodes (*n*=72), and acanthocephalans (*n*=1). Helminths recovered by necropsy in heartworm infected dogs included *A. caninum*, *A. brasiliense*, *T. vulpis*, *D. caninum*, and *Spirometra mansonoides*. Helminths recovered by necropsy in heartworm non-infected dogs included *A. caninium, A. brasiliense, T. vulpis, D. caninum* and *S. mansonoides*. Additionally, GI helminths recovered only heartworm non-infected dogs were *T. canis* and *Macracanthorynchus ingens* (Table 2). The minimum, maximum, mean intensity, and standard deviation for the intestinal helminths recovered by necropsy from heartworm non-infected dogs has been summarized in Table 3, which also includes the specificity of the DiroChek® before and after heat treatment for reference. Intestinal parasite eggs detected in the feces of dogs in this study included *Ancylostoma* spp., *Trichuris* sp., *Spirometra* and *T. canis.* Additionally, oocysts of *Cystoisospora* sp., *Sarcocystis* and *Cryptosporidium* sp. (only in 1 *D. immitis* positive dog) were found, along with *Giardia* cysts (Table 2).

**Table 2:** Overall necropsy and centrifugal Sheather’s sugar flotation results for gastrointestinal helminths or protozoa in 58 *Dirofilaria immitis* necropsy positive and 105 *D. immitis* necropsy negative dogs

*D. immitis* necropsy	Positive *n*=58		Negative *n*=105	
GI Parasites	Adult worms	Flotation (eggs/cysts/oocysts)	Adult worms	ND
Acanthocephalans				
*Macracanthorynchus ingens*	ND	ND	1	1
Cestodes				
*Dipylidium caninum*	24	ND	44	ND
*Spirometra mansonoides*	3	1	1	19
Nematodes				
*Ancylostoma caninum*	42		72	
*Ancylostoma brasiliense*	1		1	
*Ancylostoma* spp.		43		83
*Toxocara canis*	ND	ND	4	1
*Trichuris vulpis*	20	21	15	12
Protozoans				
*Cryptosporidium* sp.	N/A	1	N/A	ND
*Cystoisospora* sp.	N/A	12	N/A	19
*Giardia* sp.	N/A	ND	N/A	1
*Sarcocystis* sp.	N/A	1	N/A	1

*ND* Not Detected, *N/A* Not applicable

**Table 3:** Summary of the minimum, maximum, mean intensity, and standard deviation from mean of adult intestinal helminths in 105 dogs confirmed heartworm negative by necropsy, number of dogs with no helminths recovered but ova or proglottids detected, DiroCHEK® antigen results, specificity both with and without heat ICD

Helminth and protozoa	# Dogs	GI helminths				DiroCHEK®: # Dogs antigen positive without heat ICD	DiroCHEK®: # Dogs antigen positive with heat ICD
		Min	Max	Mean Intensity	St. dev. from Mean		
*Ancylostoma caninum*	72	1	427	44.09	64.09	0	0
*Ancylostoma braziliense*	1	4	4	4	0	0	0
*Dipylidium caninum*	37	1	61	7.05	11.54	0	0
*Toxocara canis*	4	1	2	1.25	0.5	0	0
*Trichuris vulpis*	15	1	93	9.4	25.8	0	0
*Spirometra mansonoides*	1	2	2	2	0	0	0
*Macracanthorhynchus ingens*	1	3	3	3	0	0	0
*Ancylostoma* sp. ova only	11					0	0
*Ancylostoma* sp., *T. vulpis* ova only	1					0	0
*D. caninum*: proglottids	7					0	0
*T. vulpis* ova only	2					0	0
Necropsy/flotation no parasites detected	12					0	0
						Sensitivity 100%	Sensitivity 100%

## Discussion

No cross reactivity was seen between the commercially available DiroCHEK® heartworm assay and the sera from heartworm non-infected dogs naturally infected with *Acanthocheilonema reconditum*, intestinal helminths and protozoan parasites in this study. The specificity was unchanged at 100% both before and after heat treatment assessed using serum from 105 confirmed heartworm non-infected dogs. Given the high level of parasitism in these heartworm non-infected dogs (Tables 1, 2, 3) this is an ideal population to assess potential cross reactivity of *A. reconditum* and intestinal parasites of variable infection intensities (Table 3). Indeed, the highest intensities of adult helminths observed among the confirmed heartworm non-infected dogs were 427, 61, and 93 for *Ancylostoma caninum*, *Dipylidium caninum*, and *Trichuris vulpis* respectively, none of which elicited a false positive antigen result pre- or post-ICD. Only 4 dogs had *Toxocara canis*, and additionally *Ancylostoma brasiliense, Spirometra mansonoides* and *Macrocanthorynchus ingens* were only found in 1 dog each. These parasites were not detected on the heartworm antigen tests pre- or post-heat ICD, although only a small number of dogs with these helminths were tested. It is possible that cross reactive antigens are indeed released by *A. reconditum* or by intestinal helminths at low levels but are below detectible limits. This possibility is in doubt, particularly since in this study male heartworms (6/7) were detected post heat-ICD, once thought to be undetectable due to no or low antigen released. Alternatively, if intestinal parasites do release cross reactive antigens they may be limited to the local intestinal environment, and may not enter circulation. The results of this study, in agreement with the historical literature [1, 4, 6, 12, 14, 16], found no evidence of cross-reactivity with these helminths, and thus if cross-reactions do occur it is likely very rarely.

The findings of this study, no observed cross-reactivity by *A. reconditum* post heat-ICD, agree with previous observations of previous studies using heat ICD in dogs where the heartworm status was unknown, only *A. reconditum* microfilariae were detected, and no antigen detected following post-heat ICD [3, 35]. Recently in a study involving shelter animals from Florida, USA, 2 dogs with only *A. reconditum* microfilariae, initially tested “no antigen detected” but converted to post-heat ICD antigen positive [36]. Though the true heartworm status was unknown, those 2 dogs likely had occult heartworm infections resulting in the post-heat ICD positive results.

The results reported in this study underscore the importance of using well-characterized samples by combining multiple diagnostic methods with necropsy to verify or rule out occult heartworm infections prior to assessing cross-reactivity of an organism with a diagnostic test. Previous studies have suggested that *A. reconditum* could cause false positive antigen results on heartworm antigen tests [23–27] by misciting an antibody-ELISA paper [7], and others concluded this based on interpretation of Modified Knott’s technique, antigen, post-heat ICD antigen results and compared with PCR as the gold standard reference [26], an inappropriate conclusion since occult heartworm infections nor *D. repens* were not ruled out by necropsy, the accepted gold standard [33]. Additionally, occult infections were proposed to be detected by *Wolbachia* and Filariid PCR [26], though this would require a necropsy verification of heartworm status and microfilariae testing using a filter concentration method due to its higher sensitivity versus the MKT [37]. In this study, no false positives were seen in the 8 necropsy confirmed heartworm negative dogs with *A. reconditum* identified by MKT and PCR. Another recent case report assuming cross reactivity without knowing the true heartworm status involved a previously stray dog from *D. immitis* endemic Spain, imported into the Netherlands, and suspected of heartworm infection due to microfilariae, and a post-heat ICD antigen positive test [27]. That study concluded that *Acanthochilonema dracunculoides* cross reacts with heartworm tests post-heat ICD based primarily on the post-heat ICD antigen results, PCR results, as well as echocardiography not visualizing heartworms in the trunk or pulmonary arteries, the latter a finding not uncommon in mild asymptomatic heartworm infected dogs [27]. In that report, the post-heat ICD antigen converted to “no antigen detected” approximately 3 months and 2 weeks following treatment [27]. It is possible the positive antigen results observed post-heat ICD was due to an occult *D. immitis* infection, known to test false negative on antigen tests, and as demonstrated in this study, 90.9% of false negative results testing positive post-heat ICD were due to male or female only occult infections. Male heartworms may also be a possible scenario for the initial post-heat ICD antigen positive results and short period (~104 days) to “no antigen detected” post-heat ICD, particularly due to the low level of antigen released by male heartworms [38]. The observed “no antigen detected” following the 3 month two week treatment described above is similar to results of 2 owned, heartworm infected dogs (stage 1) which converted to “no antigen detected’ at 89 days (post-heat ICD 130 days), and 90 days following a similar treatment regimen of doxycycline, and twice monthly imidacloprid/moxidectin [39]. While the possibility remains that *A. dracunculoides* does indeed cross react with heartworm tests post-heat ICD, and shown to react using standard protocols under extreme infection intensities [20] at this time it should be considered inconclusive. This possibility should be further investigated with well characterized samples that are microfilaremic for *A. dracunculoides*, and with occult *D. immitis* and *Dirofilaria repens* verified absent at necropsy, or experimental samples evaluated, similar to that recently reported for *D. repens* [21].

While the specificity observed with the DiroCHEK® in this study was unchanged at 100% both pre-and post-heat ICD antigen testing of serum, it is important to consider the animal history and clinical factors when interpreting antigen results [3]. Four of the 58 heartworm infected dogs in this study each had a single mature *D. immitis* in the thoracic cavity. One of these 4 dogs had no heartworms present in the cardiopulmonary system, only the thoracic cavity, and would have been presumably an unverifiable antigen positive by other diagnostic methods with the exception of potential eosinophilia on a CBC blood panel [39]. In another study evaluating heat-ICD of serum from necropsy confirmed samples, specificity decreased from 97.8% to 96.1 while sensitivity increased by 7.7% for mature infections [28]. In that study it was unclear if ectopic infections were present or if other factors caused the presumed false positives observed both pre-and post-heat ICD but should reinforce that no diagnostic test or method is perfect.

Given the recent reports of *D. repens* in a shelter dog and cat from a Florida, USA shelter and unknown whether those cases were imported or were acquired locally, there is a need for a multiple diagnostic approach and increased awareness and surveillance particularly among sheltered animals [36]. It has been recognized that imported or travel related introduction of parasites to non-endemic areas are not uncommon and suggested that veterinarians should consider non-endemic parasites as differential diagnoses [21]. Imported infections should be of increased concern given that the Centers for Disease Control and Prevention (CDC) estimates approximately 1.06 million dogs are imported into the United States each year [40].

Parasitic infections in North America known to cross-react with heartworm tests include *A. vasorum* and *S. lupi,* both rarely reported and often asymptomatic, can be diagnosed using different means and therefore, differentiated from *D. immits* [41, 42]. In North America, *A. vasorum* is considered an emerging threat and is endemic in wild canids of Newfoundland and Nova Scotia, Canada [41, 43] with a single case report from a red fox from West Virginia, USA [44]. *Spirocerca lupi* is endemic in wild canid populations in the southeastern US and has been reported in bobcats, grey and red foxes, and coyotes in Florida, USA [45]. In this study, although methods were included to detect these parasites, they were not recovered during necropsy of dogs and *D. repens* was not identified by MKT or PCR testing of blood. *Dracunculus insignis*is, also not found in this study is suspected to cross-react with heartworm antigen tests [3, 46]and should be further investigated regarding potential cross-reactivity with heartworm tests. Two microfilariae of an unknown species, which measured 427 × 7.6 µm and 408.6 × 7.3 µm, were found during MKT with microfilariae of *D. immitis*. Since they were present in a dog with a known *D. immitis* infection, it is not determined whether or not their presence contributed to the positive heartworm antigen result.

Limitations in this study include the use of the MKT, known to have a lower sensitivity for microfilariae versus a filtration concentration method, particularly when microfilaremia is very low [37]. Although if additional microfilariae were detected among the heartworm non-infected dogs, this would only strengthen the conclusions of this study given the pre- and post-heat ICD results. Additional parasites may have been present in this population and detection aided by using fecal sedimentation or more extensive examination of additional organs and/or soaking of the tissues. The prior history of parasite preventive in this population was unknown, although many of these dogs may have received some type of anti-parasiticide prior to euthanasia, which may have reduced recovery of intestinal parasites or had an unknown effect on potential cross-reactive antigenemia.

The overall data presented here concludes that the DiroCHEK® heartworm antigen test is unlikely to cross-react with *A. reconditum* or the intestinal parasites evaluated here with or without the use of heat ICD of serum. When evaluating potential cross reactivity of diagnostic tests, ruling out occult *D. immitis* infections, intestinal, or tissue inhabiting parasites by necropsy combined with a multiple diagnostic approach is important. Additional surveillance is needed for helminths in domestic dog and wild canid populations by necropsy and/or other diagnostics. Further research to determine potential cross-reacting organisms is important to improve heartworm antigen tests, aid development of a definitive confirmation test, and to aid in differential diagnosis. Given the high sensitivity following heat treatment for detection of mature heartworm infections demonstrated here, and the high specificity observed in this study, the use of heat treatment may allow increased confidence for detection of mature heartworm infections in dogs without fear of compromising results due to some common, often concurrent, parasitic infections.

## Conclusions

The conclusions of this study suggest that the use of heat treatment improves sensitivity of heartworm tests without false positive antigen results due to *A. reconditum*, intestinal helminths and protozoal parasites in dogs.

## Data Availability

All data generated or analyzed during this study are included in the published article.
